# Supplementary figures and images for: SARS-CoV-2 nucleocapsid protein inhibits the PKR-mediated integrated stress response through RNA-binding domain N2b
Source: PLoS Pathog. 2023 Aug 22;19(8):e1011582. doi: 10.1371/journal.ppat.1011582 (PMC10473545; doi:10.1371/journal.ppat.1011582)

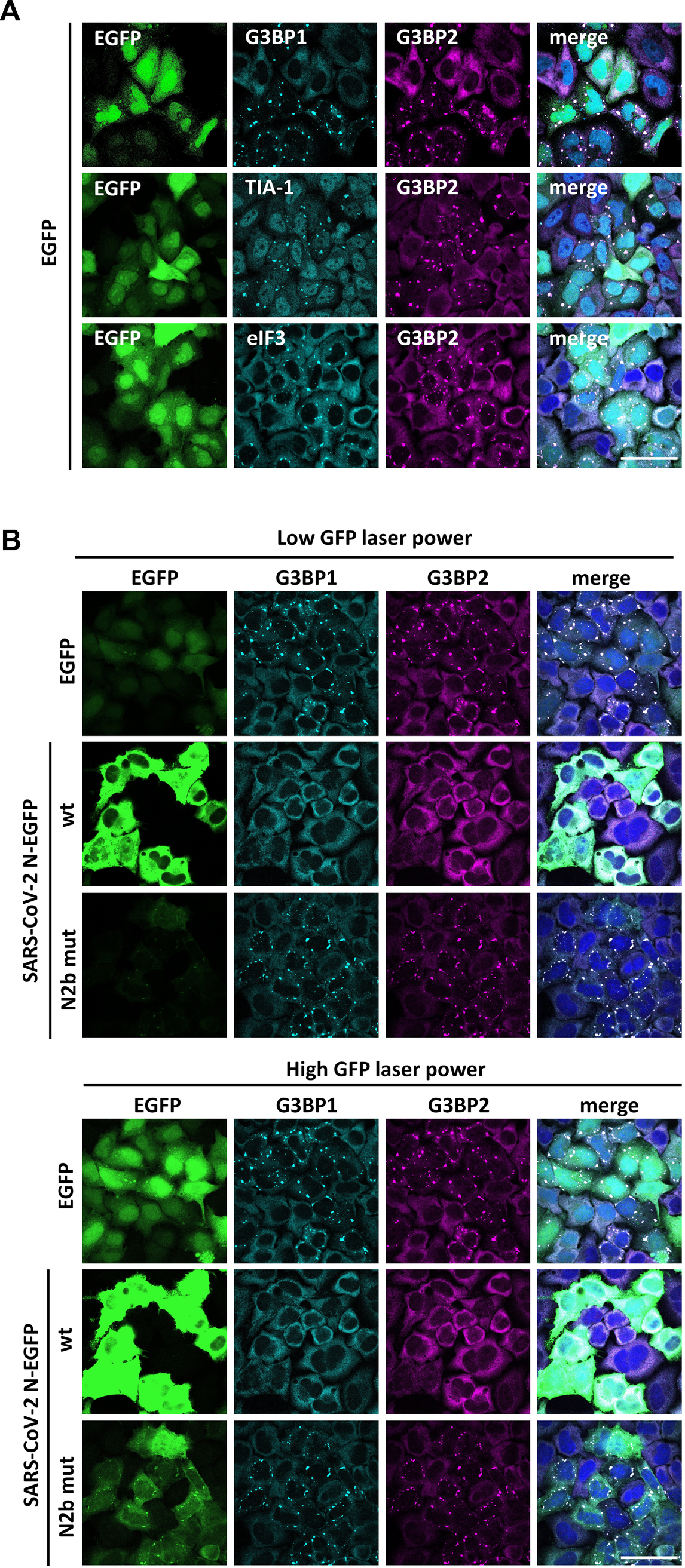

Supplement: S1 Fig — (A) Transfection with pEGFP-based transfection vectors induces SG formation. Hela wt cells, transfected with pEGFP, were stained for SG markers. Granules positive for G3BP2 also contain G3BP1, TIA-1 and eIF3. EGFP intensity digitally increased with respect to standard conditions to show all EGFP+ cells (see below). Size bar: 50 μm. (B) Cell-to-cell heterogeneity in pEGFP-driven EGFP and N-EGFP expression levels. Hela wt cells were transfected to express EGFP, SARS-CoV-2 N-EGFP or SARS-CoV-2 N-N2b mut-EGFP, i.e. a derivative unable to inhibit the ISR (vide infra). Expression levels were assessed by fluorescence microscopy as in Fig 1A. Images acquired at standard (low) EGFP intensity laser power (top) and high intensity laser power (bottom) to illustrate that all cells containing SGs are in fact transfected. Size bar: 50 μm. (TIF) [file ppat.1011582.s001.tif]

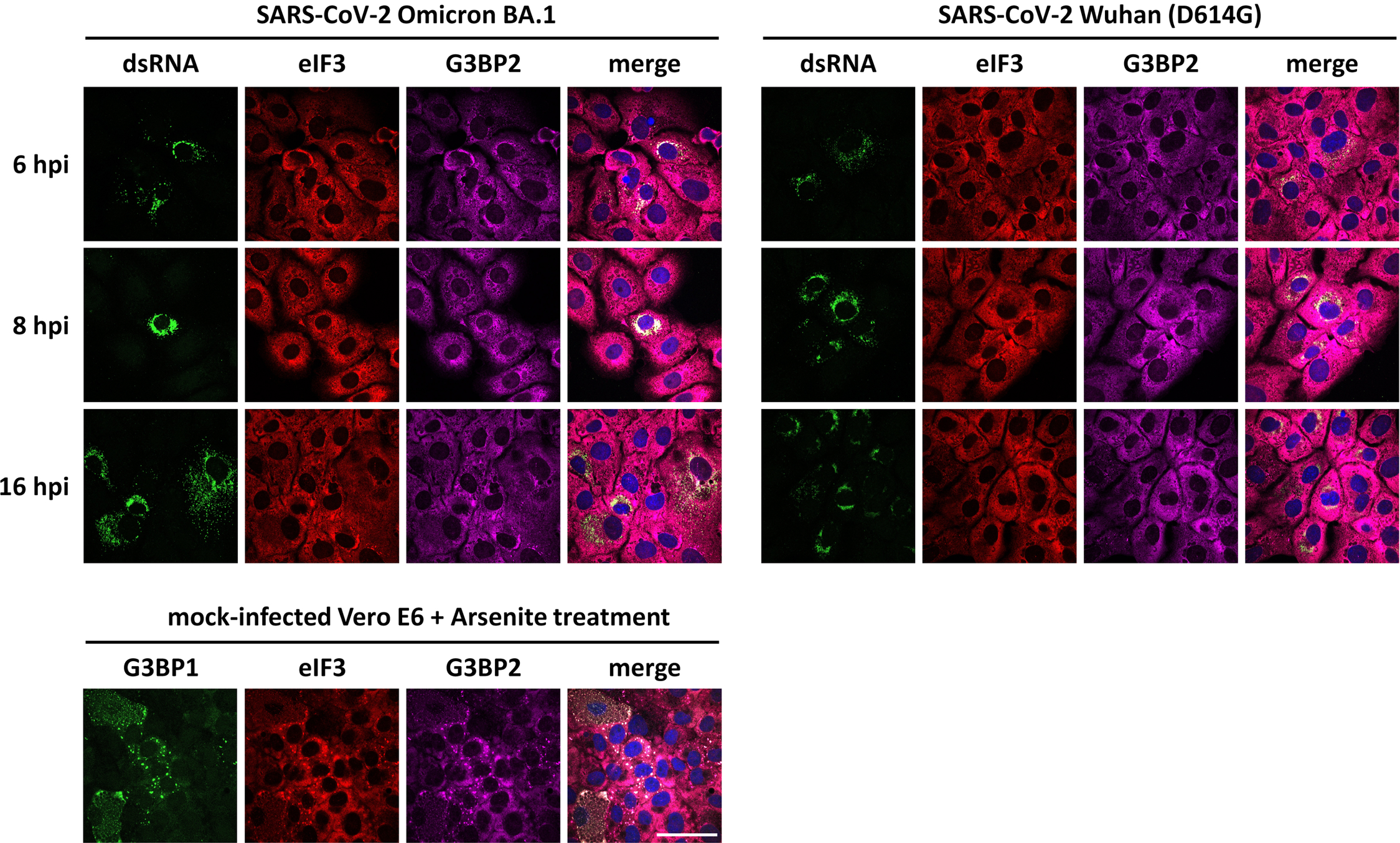

Supplement: S2 Fig — Vero E6 cells stably expressing TMPRSS2 were infected with SARS-CoV-2 Omicron BA.1 and Wuhan (D614G) variants at MOI 5. After 6, 8 and 16 hpi cells were fixed and stained with antibodies against dsRNA as an infection marker, and eIF3 and G3BP2 as SG markers. Mock-infected cells were treated with sodium arsenite to induce SGs and stained with antibodies against G3BP1, G3BP2 and eIF3. Size bar: 50 μm. (TIF) [file ppat.1011582.s002.tif]

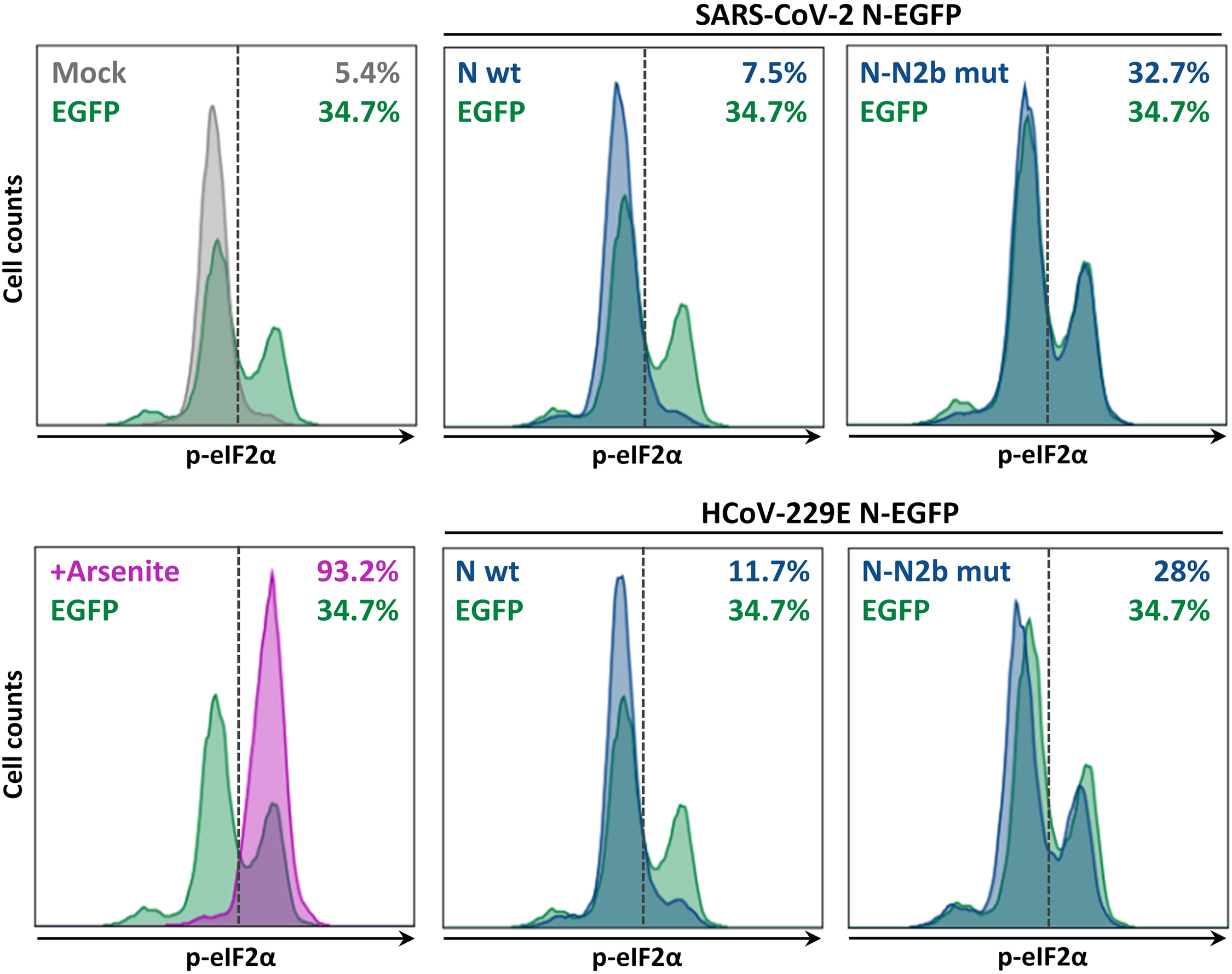

Supplement: S3 Fig — SARS-CoV-2 N and HCoV-229E N proteins prevent eIF2α phosphorylation. Hela wt cells were (mock)transfected to express EGFP, SARS-CoV-2 N-EGFP, SARS-CoV-2 N-N2b mut-EGFP, HCoV-229E N-EGFP and HCoV-229E N-N2b mut-EGFP. Phosphorylated eIF2α (p-eIF2α) levels were assessed by flow cytometry. The dashed line divides p-eIF2α negative (left) from p-eIF2α positive (right) cell populations. The percentage of positive p-eIF2α cells is indicated in the top right part of each panel. (TIF) [file ppat.1011582.s003.tif]

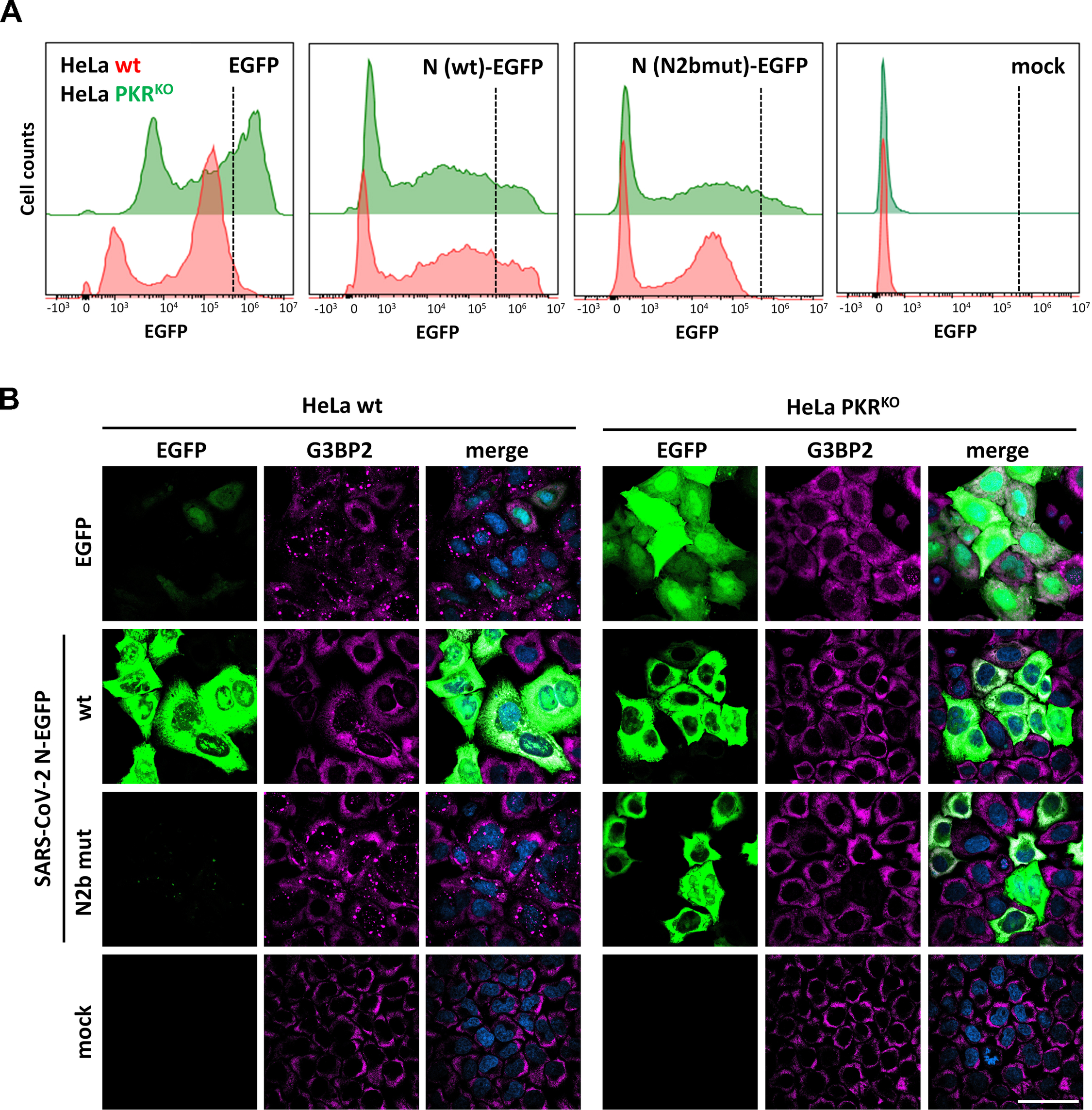

Supplement: S4 Fig — Basal expression levels of N-EGFP and EGFP. Hela wt and HeLa PKRKO cells were (mock)transfected to express EGFP, SARS-CoV-2 N-EGFP and N-N2b mut-EGFP, i.e. a derivative unable to inhibit the ISR. (A) Expression levels assessed by flow cytometry. Cell counts plotted against EGFP fluorescence intensity. The dashed line marks maximum fluorescence intensity observed in pEGFP-transfected, HeLa wt cells. (B) Expression levels assessed by fluorescence microscopy as in Fig 1A. The N-EGFP fusion protein is non-codon optimized and three times larger in size than codon-optimized EGFP. Note that expression of EGFP, as indicated by average fluorescence intensity, is higher than that of N-EGFP when compared in HeLa PKRKO cells, i.e. in the absence of translational arrest. However, also note that in HeLa wt cells, under conditions of PKR-mediated ISR-induced translational arrest, (i) expression of EGFP is restricted and (ii) as a result, expression of N-EGFP greatly exceeds that of EGFP in a sizeable population of transfected cells. The analyses have been performed for all the constructs used in this study; data available upon request. Size bar: 50 μm. (TIF) [file ppat.1011582.s004.tif]

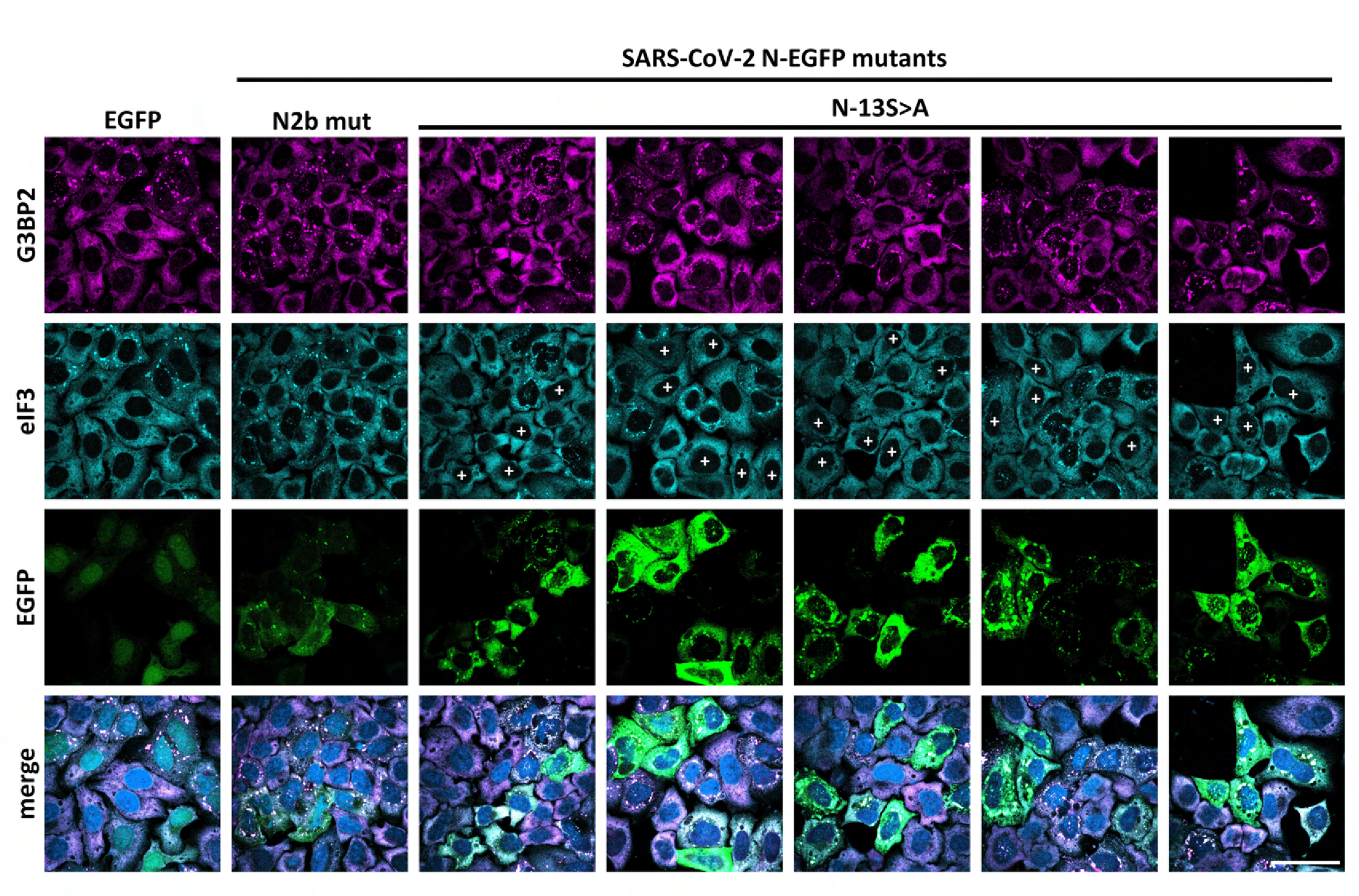

Supplement: S5 Fig — Mutagenesis of the serine rich region (SR) of SARS-CoV-2 N alters the cellular distribution of N but does not affect its ability to inhibit ISR-induced translation arrest. HeLa R19 cells were transfected to express SARS-CoV-2 N (13S>A)-EGFP. Transfected cells were stained for eIF3 and G3BP2 as markers for SGs. Rather than distributing throughout the cytoplasm, the mutant protein accumulates in large local deposits resembling aggregates. These accumulates contain G3BP2 but in most cases are devoid of eIF3 (cells marked with white crosses). Note that the fluorescence intensity in N (13S>A)-EGFP expressing cells is comparable to that in cells expressing parental N-EGFP (see e.g. Figs 1 and 4) and consistently higher than in cells expressing EGFP alone or N-N2b mut-EGFP. Size bar: 50 μm. (TIF) [file ppat.1011582.s005.tif]

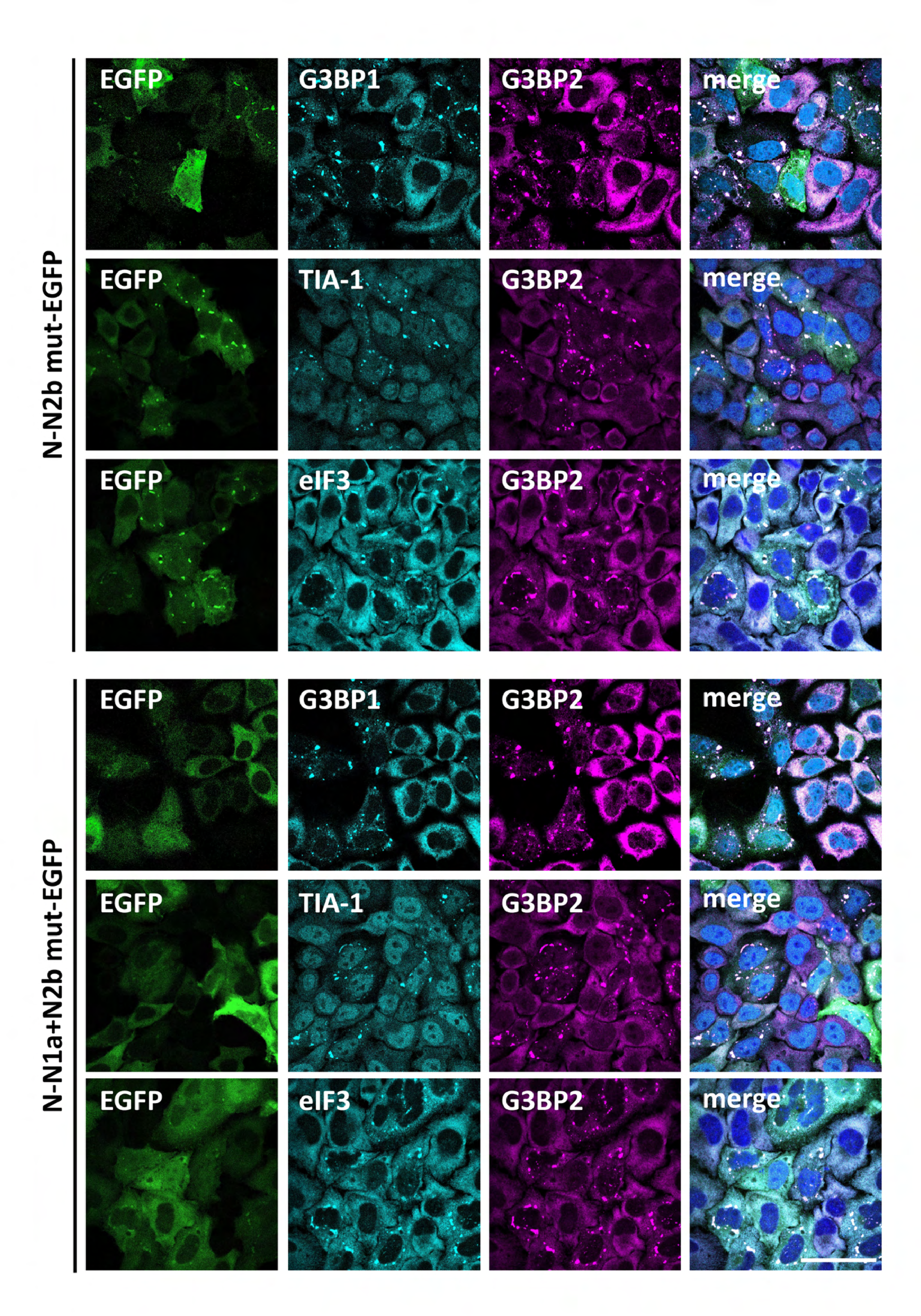

Supplement: S6 Fig — N2b-defective N-EGFP derivatives are recruited to SGs but strictly dependent on the presence of a functional G3BP binding site in subdomain N1a. Hela wt cells were transfected to express SARS-CoV-2 N-N2b mut-EGFP or SARS-CoV-2 N-N1a+N2b mut-EGFP and stained for SGs. Due to the mutations in N2b (K257A+K261A) SGs are formed in both cases, but only N-N2b mut-EGFP co-localizes with SGs. Apparently, the I14A+R15A substitutions in the ΦXFG G3BP1 binding motif in N1a abrogate SG recruitment. EGFP intensity digitally increased with respect to standard conditions to show all EGFP+ cells. Size bar: 50 μm. (TIF) [file ppat.1011582.s006.tif]

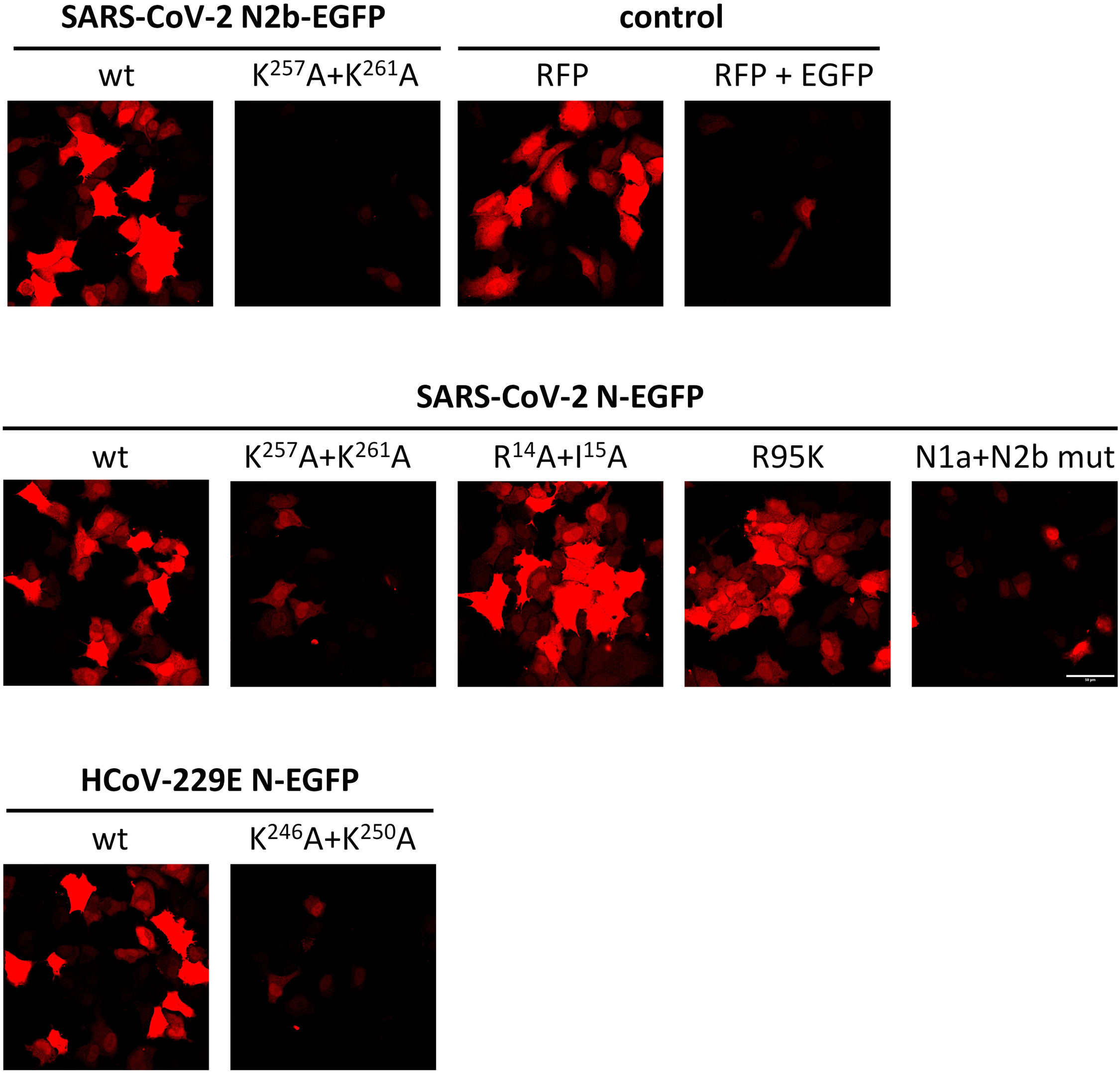

Supplement: S7 Fig — Co-transfection assay to test for translational repression in trans. HeLa cells were transfected to express EGFP, EGFP-tagged N domain N2b, EGFP-tagged full length N and mutant derivatives thereof from pEGFP-N3-based expression vectors to induce PKR-activated ISR. The capacity of these proteins to rescue translational repression in trans of red fluorescent protein (RFP), expressed from vector pcDNA-RFP, or lack thereof was measured by fluorescence microscopy. Scale bar: 50 μm. (TIF) [file ppat.1011582.s007.tif]

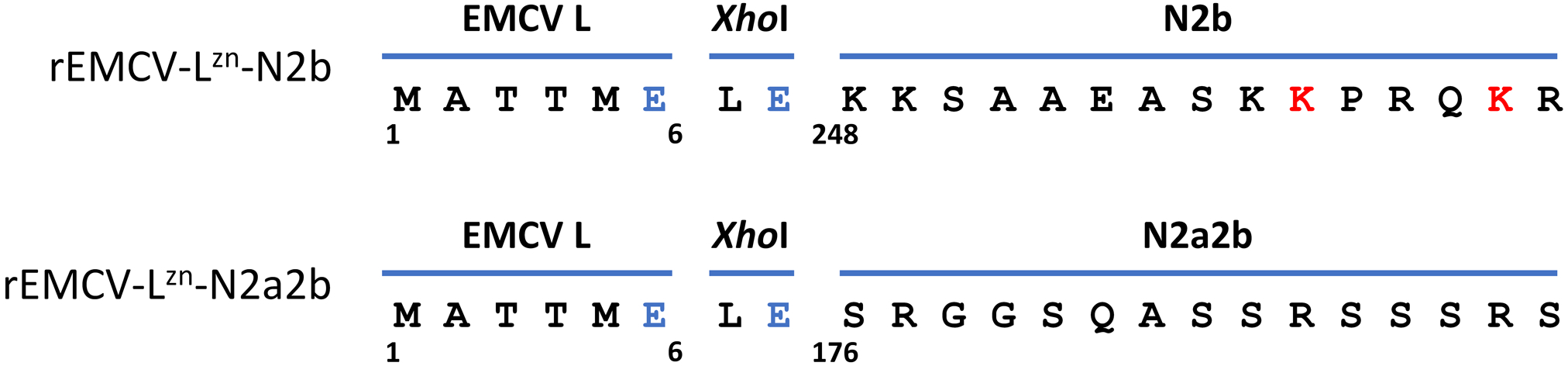

Supplement: S8 Fig — N-termini of the rEMVC-Lzn-N2b and rEMVC-Lzn-N2a2b polyproteins. Indicated are the N-terminal six residues of EMVC L, the two residues encoded by an engineered XhoI cleavage site fused to SARS CoV-2 N residues 248–365 or 176–365, respectively. (TIF) [file ppat.1011582.s008.tif]

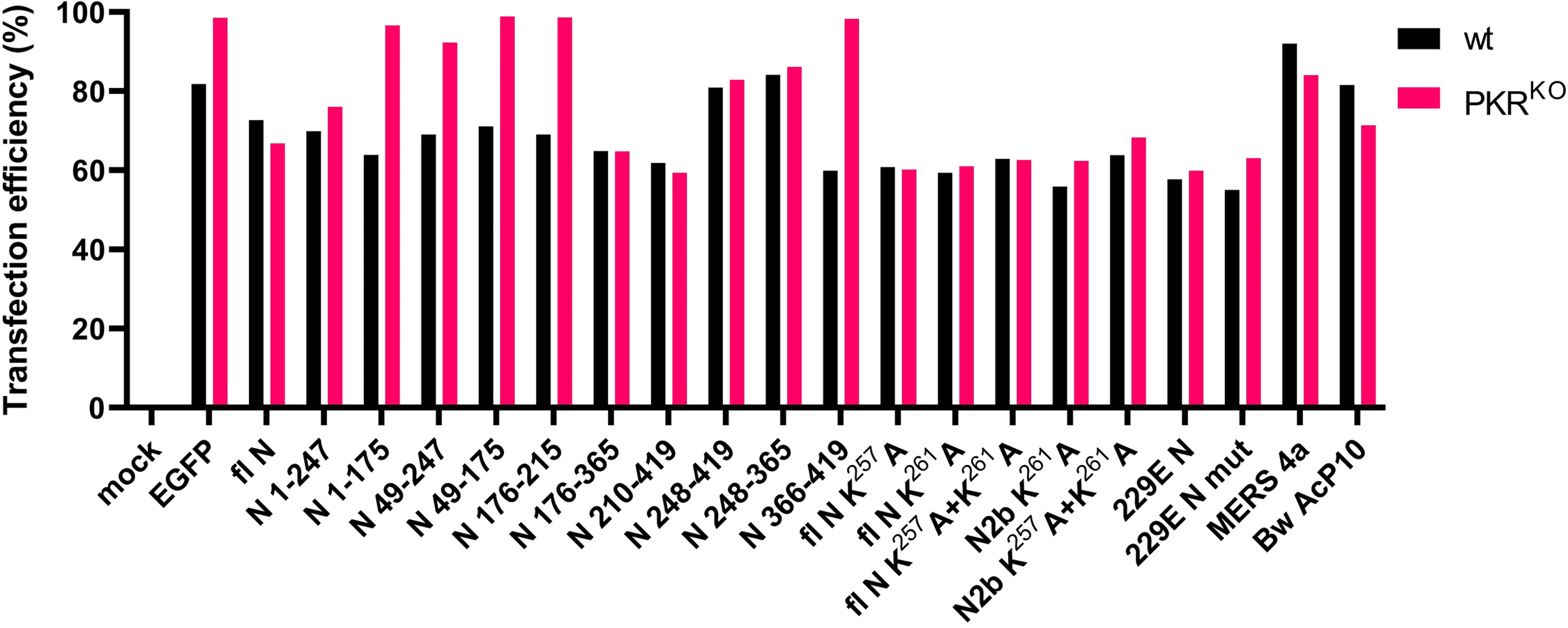

Supplement: S9 Fig — Transfection efficacy of transfected constructs used throughout this study has been measured by flow-cytometry in HeLa-R19 wt and HeLa-R19 PKRKO cells. (TIF) [file ppat.1011582.s009.tif]

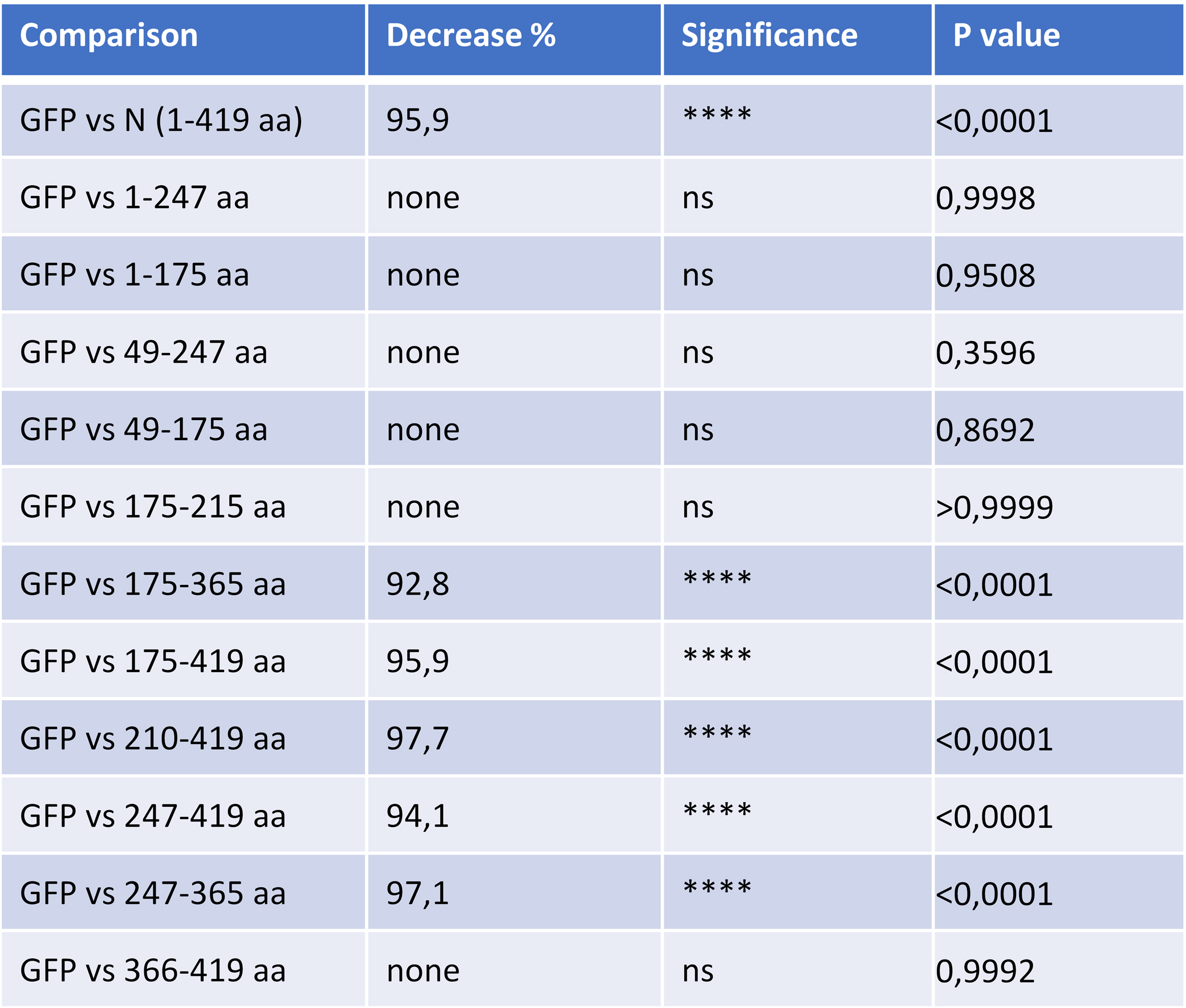

Supplement: S1 Table — Ordinary One-way ANOVA, Dunnett’s multiple comparison test. (TIF) [file ppat.1011582.s010.tif]

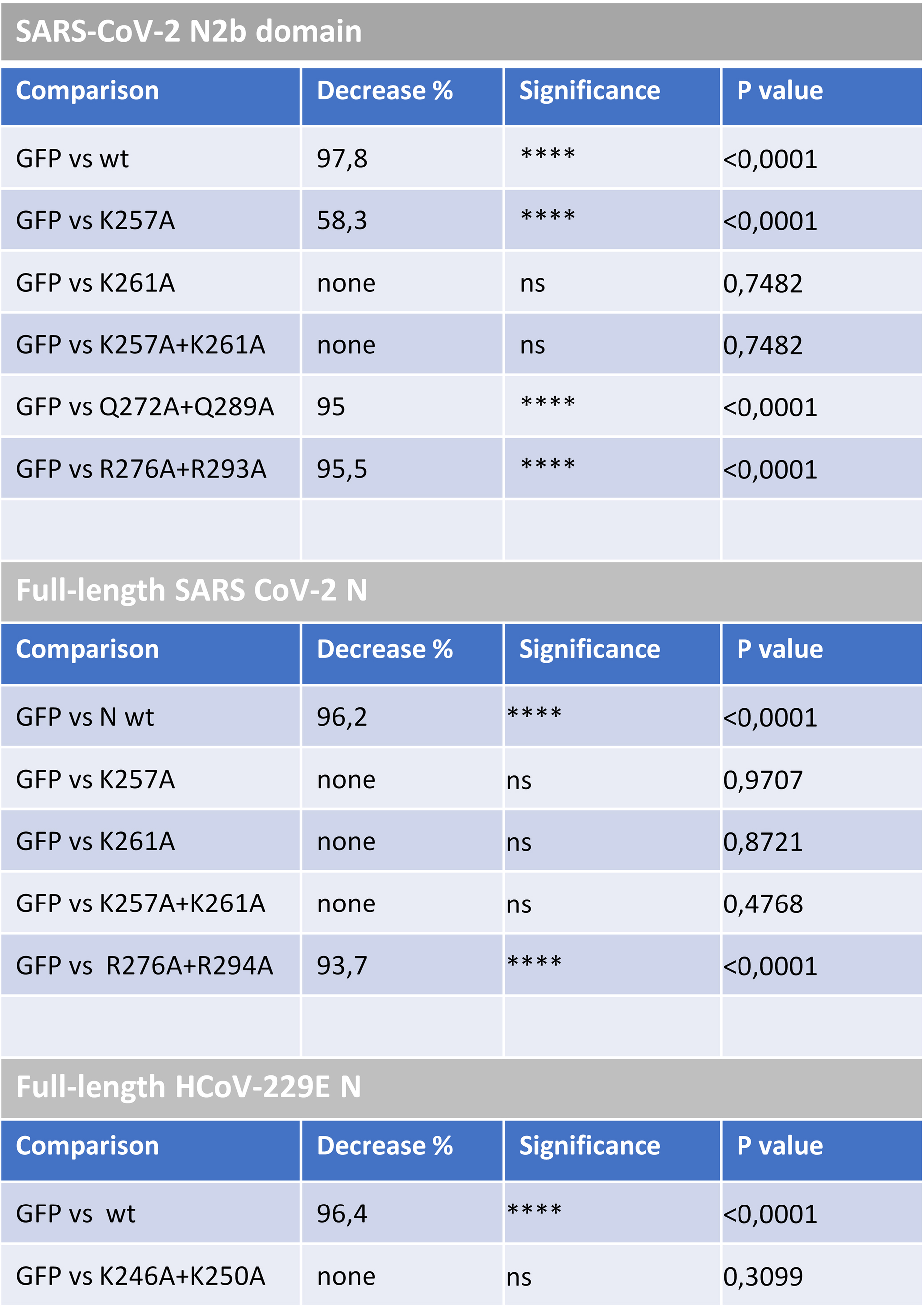

Supplement: S2 Table — Ordinary One-way ANOVA, Dunnett’s multiple comparison test. (TIF) [file ppat.1011582.s011.tif]

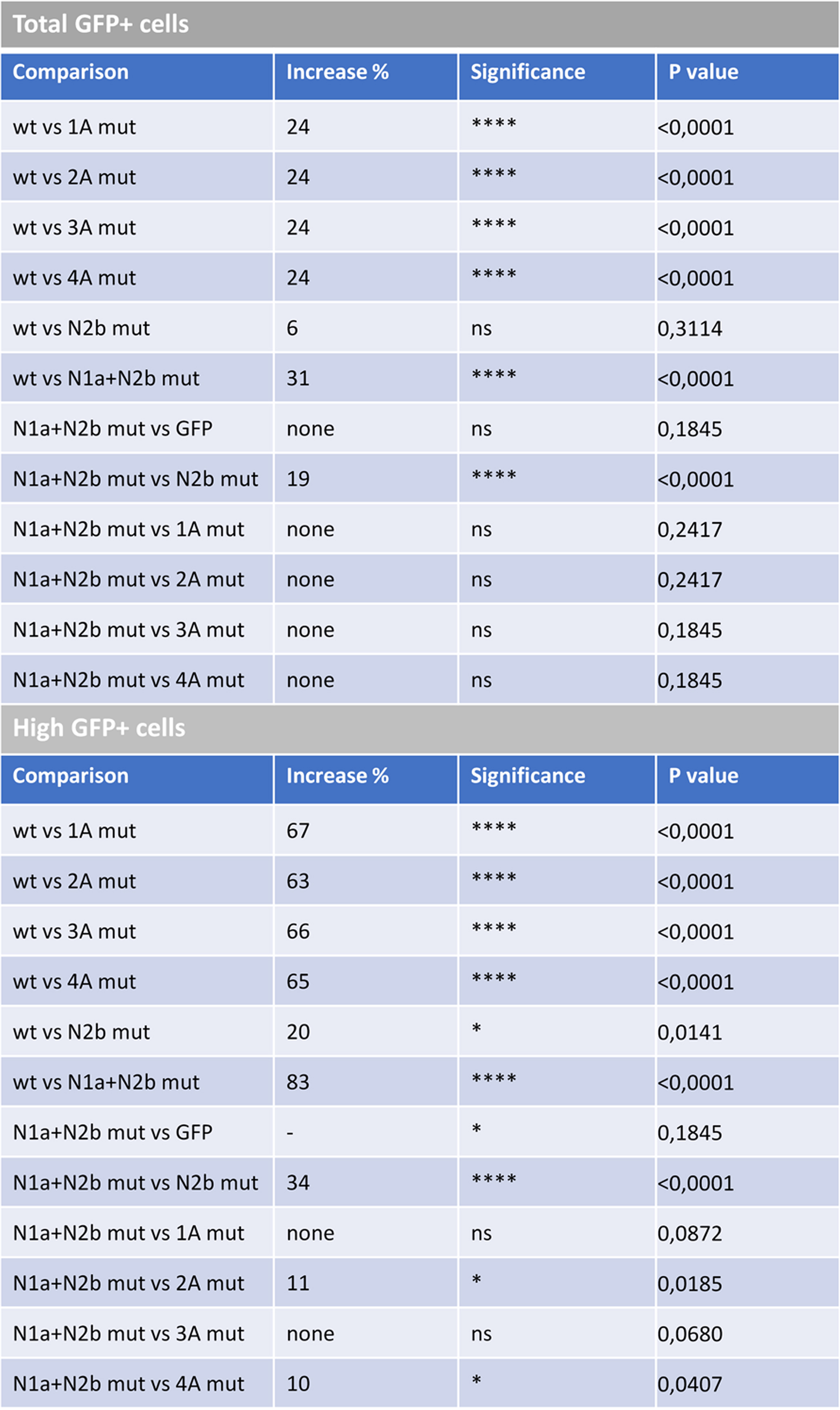

Supplement: S3 Table — Ordinary One-way ANOVA, Dunnett’s multiple comparison test. (TIF) [file ppat.1011582.s012.tif]
