# Supplementary material for: SARS-CoV-2 nucleocapsid protein inhibits the PKR-mediated integrated stress response through RNA-binding domain N2b
Source: PLoS Pathog. 2023 Aug 22;19(8):e1011582. doi: 10.1371/journal.ppat.1011582 (PMC10473545; doi:10.1371/journal.ppat.1011582)
Supplement: Attachment — Submitted filename: Resubmission letter PPATHOGENS-D-23-00424.docx [file ppat.1011582.s013.docx]

<http://www.google.nl/url?sa=i&rct=j&q=&esrc=s&source=images&cd=&cad=rja&uact=8&ved=0ahUKEwj59fWgxovNAhWCshQKHa5_AuYQjRwIBw&url=http://www.diergeneeskunde.nl/&psig=AFQjCNFQp4EaTC8LPwyDHweZtfHMTHYlqQ&ust=1465032620453704>

***Faculty of Veterinary Medicine***

**Prof. Dr. Frank van Kuppeveld**

***Head Section of Virology,***

***Division of Infectious Diseases & Immunology, Department of Biomolecular Health Sciences,***

***Faculty of Veterinary Medicine,***

***Utrecht University***

Androclus building, Room 512, Yalelaan 1, 3584 CL Utrecht, The Netherlands

Phone +31 30 253 4173

E-mail: f.j.m.vankuppeveld@uu.nl

17 July 2023

Dear Editor,

Hereby, we send you the revised version of our manuscript entitled "SARS-CoV-2 nucleocapsid protein inhibits the PKR-mediated integrated stress response through RNA-binding domain N2b" (PPATHOGENS-D-23-00424) by Aloise et al.

We thank the reviewers for their overall very positive responses as well as for their constructive comments which we have used to further improve our manuscript.

Please find in the next pages, our point-by-point responses to the comments of the reviewers.

We hope that our revised version is now suitable for publication in PLoS Pathogens.

Yours sincerely,

Prof. Frank van Kuppeveld

We are pleased to find our original manuscript was well received. As judged by Reviewer 1, *‘it comprises a methodical study of the determinants of N-mediated SG suppression, which clarifies some of the unanswered questions and confusion in the field. Experiments are carefully controlled, and the data is clearly presented. The data is high quality and provide the clearest picture to date of this property of N.*’. Reviewer 2 notes that ‘*the manuscript is very well-written, applies solid methodology and highlights the novelty of the SARS-CoV-2 and, in parts, HCoV-229E N-dependent ISR effect in the context of other published articles on SARS-CoV-2 N.*” Reviewer 3 states that ‘the data obtained adequately support for the authors’ conclusion that the N protein N2b domain binding to dsRNA and preventing PKR activation and SG formation is the primary mechanism involved.’

We are grateful for the comments and constructive criticisms by the Reviewers, which have helped us to further improve our manuscript. Please find our point-by-point response below.

**Part I - Summary**
Please use this section to discuss strengths/weaknesses of study, novelty/significance, general execution and scholarship.

**Reviewer #1:** Multiple groups have shown that coronavirus N proteins inhibit stress granule (SG) formation, but the precise mechanism remains obscure, with most studies quite focused on physical interactions with G3BP proteins. Here, Aloise, C., et al. conduct a methodical study mapping the determinants of N-mediated SG suppression. They show the N2b (aka CTD) domain that is involved in RNA binding and N dimerization is sufficient for SG suppression whereas other domains were dispensable. Substitution of key lysine residues implicated in RNA binding inactivated the N2b domain (both for SARS-CoV-2 N and hCoV-229E N), whereas substitution of key glutamine and arginine residues linked to dimerization did not affect SG formation. Thus, RNA binding appears to be central to the SG suppression mechanism. By contrast, substitution of key residues involved in N acetylation and arginine methylation showed that these PTMs are not involved in SG suppression. Similarly, the amino-terminal G3BP binding domain was dispensable for PKR-induced SG formation. Finally, citing the challenges in making and rescuing CoVs with mutations in N, the authors engineered a recombinant EMCV where the SG antagonist (L) is replaced by a portion of SARS-CoV-2 N encompassing N2a and N2b. This recombinant virus suppressed SG formation, and mutating the same key lysines involved in RNA binding reversed this phenotype.

Overall, this manuscript comprises a methodical study of the determinants of N-mediated SG suppression, which clarifies some of the unanswered questions and confusion in the field. Experiments are carefully controlled and the data is clearly presented. The data is high quality, and provide the clearest picture to date of this property of N.

**Reviewer #2:** The paper by Aloise et al. describes a detailed functional assessment of the SARS-CoV-2 N protein and its interference with the ISR. The authors show that binding of N to dsRNA is critical to inhibit the PKR-dsRNA initiation of the ISR.
The manuscript is very well-written, applies solid methodology and highlights the novelty of the SARS-CoV-2 and, in parts, HCoV-229E N-dependent ISR effect in the context of other published articles on SARS-CoV-2 N.

I have several comments mainly related to the dynamics of the SG formation and the specificity of SG detection in the submitted manuscript.

**Reviewer #3:** This study investigates the mechanisms by which the SARS-Cov-2 nucleocapsid (N) protein inhibits induction of the integrated stress response (ISR) and stress granule (SG) formation. Four previous studies showed that the SARS-CoV-2 N protein inhibited SG formation through RNA binding and interaction with G3BP. The authors first show that the expression of EGFP from a pEGFP-N3 plasmid is low and that SGs are induced. When EGFP is fused to a viral protein that is an ISR inhibitor, the expression of EGFP is much more efficient and SGs do not form. The authors previously used this protocol to analyze ISR inhibition by the AcP10 and 4a proteins of other coronaviruses. In the current study, SARS-CoV-2 N fused to EGFP enhanced translation as efficiently as proteins from 3 other viruses known to inhibit the ISR. Also, EGFP expression was enhanced in PKR KO cells in the absence of an N fusion protein indicating that the decreased expression of EGFP from the plasmid is PKR mediated. The N proteins of both SARS-CoV-2 and the 229E coronavirus inhibited PKR phosphorylation. However, Western blot data confirming N mediated inhibition of eIF2α phosphorylation were not included. Individual N protein domains were next tested for their involvement in inhibition of the ISR. Only the 2nd ordered domain (N2b) was found to be required for ISR inhibition. The two ordered domains were previously suggested to be RNA binding domains involved in nucleocapsid assembly. N also has a known N-terminal G3BP binding site and an SR region that can be phosphorylated. Based on the crystal structure of an N2b dimer, mutations in the proposed RNA binding cleft were designed and the mutant N2bs were tested for their ability to inhibit the ISR. N2b domains with single and double AA mutations in residues proposed to be involved in RNA binding inhibited RNA binding and did not suppress the ISR. Neither mutation of 13 or the 14 SR phosphorylation sites or the acetylation site affected the ability of N to suppress the ISR. Mutation of 1 to 4 residues in the G3BP binding site also did not affect the ability of N to inhibit SG formation in the context of the PKR signaling pathway. However, the effect of these mutations on the N-G3BP interaction was not analyzed. The involvement of an N-G3BP interaction in inhibition was next analyzed in PKR KO cells with SGs induced by arsenite treatment. Some reduction in the % of cells with SGs was observed when the G3BP binding site was mutated suggesting that the interaction of the N1a region with G3BP might have a partial role on reducing SG formation. Flow cytometry assay of dual fluorescence reporters was used to confirm that the N2b domain is mainly responsible for the suppression of the PKR-induced ISR. Finally, the ability of SARS-CoV-2 N2b expressed from the 5′end of the EMCV polyprotein to suppress SG formation was investigated and it was found to be almost as efficient as the EMCV L protein.

The SARS-CoV-2 N protein has many functions and the involvement of each of the known functional domains in suppression of the ISR and SG formation was investigated. Previous studies showed that the SARS-CoV-2 N protein was an inhibitor of SG formation and an RNA sensor antagonist. The present study confirmed and extended the previous observations using multiple techniques and also showed that the N protein of 229E had the same mode of action. The contributions of each of the N domains was tested. The data obtained adequately support for the authors’ conclusion that the N protein N2b domain binding to dsRNA and preventing PKR activation and SG formation is the primary mechanism involved. The authors’ data did not support previous data suggesting that acetylation of Lys 175 is required for N RNA binding or that substitution of this residue reduces N suppression of the ISR. The authors’ data also did not support previous data suggesting that the N-G3BP interaction was the main mode of SG inhibition by N. However, the author’s conclusions about N mediated reduced induction of Type I IFN and statements about suppression of cellular RNA sensor activation by N2b binding to dsRNA were not supported by any data included in the manuscript.

**Part II – Major Issues: Key Experiments Required for Acceptance**
Please use this section to detail the key new experiments or modifications of existing experiments that should be absolutely required to validate study conclusions.

Generally, there should be no more than 3 such required experiments or major modifications for a "Major Revision" recommendation. If more than 3 experiments are necessary to validate the study conclusions, then you are encouraged to recommend "Reject".

**Reviewer #1:**

The use of recombinant EMCV expressing a portion of N as a tool to investigate SG suppression during infection is a clever approach. Others have expressed N in trans to complement a N-deficient SARS-CoV-2 (Leo L, et al. Science Bulletin, 2021) and many reverse genetic systems have been developed to generate mutant CoV genomes with precise mutations (BACmid, TAR, CPER, etc.). It is undeniable that N has many important functions in CoV infection and the virus cannot replicate without it, but substituting one key lysine in N involved in SG suppression is a fairly conservative approach that could help us understand the threat that SGs pose to CoVs and corroborate findings from the ectopic overexpression studies. This is a valuable experiment that is well within reach. If a SARS-CoV-2 or hCoV-229E virus with a K257A substitution in N causes SG formation during infection and fails to replicate, then this considerably strengthens the case of these investigators.

The suggested approach looks straightforward and within reach, but only deceptively so. After receipt of the Reviews, we obtained preliminary results using our biosafe working model mouse hepatitis virus (MHV). We confirmed that the MHV N protein like those of SARS-CoV-2 and HCoV-229E, inhibits PKR-induced ISR activation and that the orthologs of SARS-CoV-2 K257 and K261 are involved. We refrained from mutating the K^257^ ortholog in the virus, because of our EMSA results in Fig. 3 showing that its substitution for Ala gives an intermediate phenotype with respect to RNA binding. Thus, negative results obtained with such a recombinant virus would be inherently inconclusive. Unfortunately, as we predicted, an MHV mutant with Ala replacing the MHV K^261^ ortholog could not be salvaged in three independent, well controlled trials.

Evidently, one might proceed and attempt salvaging this viral mutant in a supporting cell line, but this would take substantially longer than the time allotted for resubmission. Moreover, there are the issues of redundancy and cell type dependency to be considered, as coronaviruses often encode more than one antagonist of the ISR (Kindler et al. (2017) PLoS Pathog; Gao et al (2021) PLoS Pathog; Comar et al, (2022) PNAS; Aloise et al. (2022) Curr Opin Immunol), and the requirement for ISR antagonism may vary among different cell types (Banerjee et al. (2014) mBio; Li and Weiss (2016) J Virol; Kindler et al. (2017) PLoS Pathog ; Kim (2020) Front Microbiol; Li et al. (2021) PNAS), complicating matters considerably. Salvaging an N mutant virus alone as well as in combination with inactivation of other proposed MHV ISR inhibitors and testing these viruses on a variety of cells lines, would take several months, if not longer, to complete.

Most importantly, we did provide conclusive proof of principle for the capacity of CoV N proteins to inhibit the ISR during viral infection in the present study by using a less complicated viral system. Picornavirus EMCV lacks redundancy in anti-host defense such that ISR antagonism can be abolished through the inactivation of a single protein. Given that the N protein is the most abundantly expressed during CoV infection, it is reasonable to assume that it participates in countering the ISR also under natural conditions. During which phase of the infection, to what extent in relation to the activity of other virus encoded antagonists like nsP15 and in which host cells is clearly a topic for future research.

**Reviewer #2:**

1. SG formation is a very complex and dynamic process and can also be transient during virus infections. In this regard I have several remarks:

1a) Although the applied plasmid-based SG overexpression system has been published before, I would suggest to provide more information on the SG dynamics. Did the authors try different time points with and without different stressors/ different nucleic acid stimuli including SARS-CoV-2 RNA/ virus infections to identify the optimal timing and conditions for SG formation and detection?

We are aware of the complexities and dynamic nature of SG formation. With respect to the experiments in which PKR-dependent SG formation was induced by plasmid transfection, we indeed build on several previous studies in which the system was established and optimized. When aiming to discover viral antagonists of the stress response, a binary outcome -SG formation or inhibition thereof- suffices and the dynamics of SG formation are less important. The procedure used in current manuscript has been validated with well-established ISR antagonists like influenza ns1, which in turn allowed us to unequivocally identify MERS CoV ns4a (Rabouw et al. (2016) PLoS Pathog) Beluga whale coronavirus acP10, aichivirus L protein (Rabouw et al. (2020) Nat Microbiol) and, in the present study, coronavirus N protein as inhibitors of the stress response.

We concur, however, that the dynamics of SG formation and the transient nature of SGs may be of importance during viral infection. Exactly for this reason, SG formation was assessed in SARS-CoV-infected cells at different time points -6, 8 and 16 hr pi- the earliest time point coinciding with the advent of sgmRNA synthesis and N protein expression (**Fig. S2**). Also, in the case of infection with recombinant EMCVs analyses were performed at 6 and 8 hr pi. (**Fig. 7**).

1b) The source of the stress (pEGFP-N3 transfection) used to induce SG formation seems quite artificial to me. The authors should explain in more detail why the transfection of this plasmid is enough to induce the SG formation and how this reflects the “natural” infection-induced SG formation. The citations do not go back to a primary source showing this phenomenon as far as I can tell. They could cite for example https://journals.plos.org/plosone/article?id=10.1371/journal.pone. 0043283

We actually did refer to literature describing this phenomenon, even predating the Nejepinska 2012 paper (see Mellits et al. (1990) NAR; Terenzi et al. (1999) NAR). We also referred to a paper by Gantke et al. (2013), who showed that plasmid induced PKR-mediated translational inhibition can be salvaged by viral PKR antagonists. Nevertheless, this point is well taken. To clarify the mechanism of plasmid-induced activation of the ISR with ensuing formation of SGs, we now also cite Nejepinska at al. (2012) and have expanded the text on l. 123-132 to read:

*‘Transfection of cells with specific expression plasmids like pEGFP-N3 triggers the ISR through dsRNA-mediated PKR activation, resulting in translation arrest and the formation of SGs (****Fig. 1A****) [27, 41-43]. The dsRNA arises from spurious bidirectional transcription of plasmid sequences [44] and can be readily detected in transfected cells with dsRNA-specific antibodies [27]. This phenomenon allows for a convenient method to identify potential viral ISR antagonists by transiently expressing these proteins genetically fused to enhanced green fluorescent protein (EGFP) [27, 31]. The expression levels of these fusion proteins, as judged by fluorescence microscopy, can then be compared to that of EGFP alone as an indicator for translation arrest and the prevention thereof.’*

1c) There are several instances where SGs appear in seemingly untransfected cells e.g. Fig 1B EGFP, Fig 2B EGFP, 366-419, Fig 3 EGFP, K257A, Fig 4A EGFP, N1a + N2b mut, Fig 5 EGFP, 1A mut, 2A mut, 3A mut. Is this regarded as background noise or might there be unwanted stimulations e.g. by endotoxins in the plasmid preps?

This is not background noise but results from differences in EGFP expression levels in cells in which the stress response remains intact (please note that the expression products mentioned are all unable to prevent activation of the stress response). Due to cell-to-cell heterogeneity, EGFP expression varies from one transfected cell to the other. For the preparation of the figures, image acquisition (laser intensity, exposure time) and processing conditions were kept constant to allow for fair comparison and chosen to avoid over-exposure in high-expressing cells. Under these conditions, EGFP expression is too low to be detected in a sizeable population of stressed cells. When images are captured at higher laser intensity or when image brightness is adjusted manually, it becomes clear that in fact **all** cells with SGs express EGFP and hence were transfected successfully. An additional supplemental figure is included to clarify the issue (**Fig. S1B**).

1d) In Fig 1B the authors show a striking difference in the GFP levels between cells transfected with pEGFP-N3 and several viral genes/orfs that are expressed as fusion proteins with EGFP-N3. SARS-CoV-2 N, MERS-4a and IAV-NS1 increase the GFP levels immensely, which the authors show to be due to inhibition of the integrated stress response (shown by lack of SG formation and absence of PKR phosphorylation). As a control they also transfect PKR knockout cells in which the difference is abrogated. In a GFP Western blot they show unexpected protein level differences between the ISR-antagonizing proteins, which they regard as an artefact possibly caused by differences in baseline transcription due to lack of codon optimization of the fused protein. They do not address, however, the discrepancy of protein levels between WB and IFA in the HeLa wt cells, where GFP-N and GFP-NS1 show much higher fluorescence but seemingly lower protein levels. Please mention in more detail how this can be explained.

We indeed consider this an artefact. Please note that the explanations given in the Legends to **Fig. 1** also hold for the observations in the HeLa wt cells. To clarify this, we have adapted the text to read: ‘*the larger yield of EGFP versus N-EGFP in either cell type is counter intuitive*’.

Importantly, we consider differences in baseline translation levels (related to codon composition *and* protein size) a potential cause, but not exclusively. We also explain that Western blot analysis entails as cited ‘*an ensemble measurement (for the total transfected cell population) versus the analysis of individual cells by fluorescence microscopy*.’ Immune fluorescence reveals a sizeable population, but subpopulation nonetheless, over-expressing the EGFP-tagged viral ISR antagonists. There is another population of transfected cells, in which the ISR is inhibited (as indicated by the absence of SGs) and in which the baseline expression levels are as low or even lower that those expressing EGFP alone (for reasons stated above) even when in these latter cells the ISR becomes activated (see also the results of flow cytometry in **Fig. S4A**). A plausible reason for seeing low proteins levels of IAV NS1 and BW AcP10 in Western Blot is that selective loss in cellular debris may have occurred due to the use of cleared, ‘post-nuclear’ lysates. IAV NS1 shuttles to the nucleus (Greenspan et al. (1988) J Virol; Melén et al. (2007) J Virol) and AcP10 forms large aggregates (Rabouw et al. (2020) Nat Microbiol). We do not have a single definite explanation but do address the phenomenon briefly as not to disrupt the flow of the main text or legend. The fact remains that these results do not jeopardize our overall conclusions that are based on a several independent lines of evidence obtained with different complimentary experimental techniques.

2. The use of a single SG marker (anti-G3BP2 antibody) is in my opinion not sufficient. The authors should clarify how specific the staining is by comparing it with other SG markers (e.g. TIA-1).

The Reviewer raises an important point and we in fact concur. At the onset of the study, we ascertained that the granules induced by plasmid transfection stained positive for G3BP1, G3BP2, eIF3 and TIA-1. To clarify this, a new supplemental figure (**Fig. S1A**) has been included. In the current manuscript, we mostly show the results obtained with G3BP2 but in our experiments routinely took monolayers stained for eIF3 along as a control.

This is particularly important as G3BP proteins can directly interact with the nucleocapsid proteins.

N proteins indeed interact with G3BPs as also confirmed in current study. In transfected cells expressing N-EGFP derivatives with mutations in N2b, G3BP-positive granules are formed, irrespective of N-G3BP association. When the N1A G3BP binding motif is left intact, the protein co-localizes with SGs, but not when the binding motif is abolished in addition. The results are included as an additional supplemental figure **Fig. S6**. These findings, together with those shown in the new supplemental figure **Fig. S1A,** conclusively show that the granules observed are not N-G3BP aggregates but genuine SGs.

**Reviewer #3:**

(*from the Summary*)

The N proteins of both SARS-CoV-2 and the 229E coronavirus inhibited PKR phosphorylation. However, Western blot data confirming N mediated inhibition of eIF2α phosphorylation were not included.

Point well taken. We have expanded **Fig. 1** to include a new panel **F**, showing activation of eIF2α by WB analysis in pEGFP-transfected cells, but not in cells expressing SARS-COV-2 N-EGFP or 229E N-EGFP. These observations were corroborated with a more quantitative, flow cytometric analysis of eIF2α activation or lack thereof now provided in a newly added **Fig. S3**.

The effect of N mutations on the N-G3BP interaction was not analyzed.

*See also the Reviewer’s comment from the Summary*:

Mutation of 1 to 4 residues in the G3BP binding site also did not affect the ability of N to inhibit SG formation in the context of the PKR signaling pathway. However, the effect of these mutations on the N-G3BP interaction was not analyzed.

We actually did analyze the effect of the mutations on N-G3BP interaction by performing pulldown experiments. Please see **Fig. 4C**. We showed N-G3BP co-precipitation and loss thereof upon mutagenesis of the G3BP1 interaction motif in N domain N1a. We also showed that mutations in N2b that abolish RNA binding do not affect N-G3BP1 association. In the revised manuscript, we present additional independent evidence from IFA in newly added **Fig. S6**.

Although the title of the final Results section indicates that the effect of N2b on the Type 1 IFN response was also tested, no Fig.7 E panel showing these data were included. Also, no data confirming the statement that this effect was due to reduced activation of RIG-I were included.

*See also the Reviewer’s comment from the Summary:*

The authors’ conclusions about N mediated reduced induction of Type I IFN and statements about suppression of cellular RNA sensor activation by N2b binding to dsRNA were not supported by any data included in the manuscript.

We are genuinely surprised by this remark. The **Fig. 7E** panel (please see below) *was* included in the original manuscript, showing (i) inhibition of IFN-β expression in wt EMCV-infected cells, (ii) loss thereof in a mutant in which the EMCV antagonist of innate immunity (L) is inactivated (EMCV-L^zn^), (iii) inhibition of IFN β expression in a mutant in which EMCV L is replaced by the SARS N2aN2b protein (noticeable both at 6 and 8 hr pi) (N2aN2b) and (iv) loss of this activity when N2b-mediated dsRNA binding is abolished by mutations (N2aN2b-Mut), noticeable particularly at 8 hr pi for reasons explained in the text.

Retinoic acid-inducible gene I (RIG-I)-like receptors (RLRs) are key sensors of viral RNA, mediating the transcriptional induction of type I interferons through activation of major IFN transcription factor IRF3 (Dias Junior et al. (2019) Trends Microbiol; Rehwinkel and Gack (2020) Nature Rev Immunol; Onomoto et al, (2021) Cell Mol Immunol). To strengthen our findings and conclusions, we include new data in panel **Fig. 7D** to show that RLR-mediated activation of IRF3 phosphorylation is inhibited in cells infected with wt EMCV or EMCV-N2aN2b but not in cells infected with EMCV-L^zn^ or EMCV-N2aN2b-Mut. On a final note, we do not make explicit claims about RIG-I specifically (as might be inferred by the Reviewer’s comment). In fact, we consistently use the generic term ‘RLRs’ to also include RLR family members like MDA5, which is known to recognize picornaviruses (which lack 5'PPP and instead contain VPg at 5'end of their genome) (Feng et al. (2012) Cell Rep). We feel that we draw our conclusions appropriately cautiously (for example see l. 434-436: ‘*Our findings suggest that N, through the autonomous activity of the N2b domain, acts not only as a classical PKR inhibitor to thwart ISR activation but may also prevent RLR activation and thus block induction of the type I IFN response.*’). We therefore stand by our findings and our statements.

**Part III – Minor Issues: Editorial and Data Presentation Modifications**
Please use this section for editorial suggestions as well as relatively minor modifications of existing data that would enhance clarity.

**Reviewer #1:**

Additional comments:

Figure 3A. The description of N structure in the text is quite informative. Can the cartoon model be improved to match this description to help the reader appreciate these details?

We have revised **Fig. 3A** to match the description in the main text and adapted the legend accordingly.

Figure 3D. Testing the Q272A+Q289A mutants and the R276A+R293A mutants was a good idea to more tightly link the phenotype to RNA binding, but since it is stated that these residues are involved in the dimer interface, it should be feasible to demonstrate that dimers do not form properly when these substitutions are made. The citation alone is not sufficient.
The specific aim of these experiments was to identify residues essential for RNA binding, the substitution of which would lead to loss of N-mediated ISR inhibition. Indeed Lys^257^ and Lys^261^ were replaced to this effect. Both residues are located in a positively-charged groove postulated by others to be involved in RNA binding (Chen et al. (2007) J Mol Biol; Takeda et al. (2008) J Mol Biol; Zhou et al. (2020) Mol Biomed; Chang et al. (2014) Antiviral Res). To go the extra mile and test whether other surface-exposed charged/polar residues, located outside the groove, would also affect binding of RNA, we introduced Q^272^A, Q^289^A, R^276^A and R^293^A substitutions. These residues have been implicated -though not conclusively- in higher order dimer-dimer interactions as based on N2b crystal packing (Chen et al. (2007) J Mol Biol) with results of disulfide trapping assays in support (Chang et al. (2013) PLoS One; Yang et al (2020) Front Chem). The debate on their importance in higher order complex formation, however, has not been settled definitively and it is not our intention to enter this complex discussion, certainly not in a manuscript uniquely focusing on a role of N in innate immunity as a potential dsRNA-binding PKR antagonist. The suggested experiments are not only complicated but, in our view, not within the scope of current study.

There is a lot of discussion of how interfering with SG formation influenced the evolution and expression levels of sarbecovirus N proteins, but this is quite speculative.

This is indeed a speculative topic, on which we spend a few words, though not too many and in the Discussion section, where some speculation is allowed (l. 408-415). Kindly take into account that we refer to recent findings and conclusions by others (Thorne *et al.* (2022) *Nature* **602;** Leary *et al.*, (2021) *Pathog Immun* **6**), which would be inappropriate to ignore.

**Reviewer #2:**

1. In some figures only SE-bars are shown, while in some there are data points. I think it would be preferable to show data points in all Figures.

Figs. were adapted according to the recommendations of the Reviewer.

2. Representing number of SG per cell rather than SG-positive cells would allow a better quantitative analysis of the implication of N2b domain in SG formation. For example, the paper by Kruse et al. detected intensity of G3BP1 foci instead of % positive cells. Using this as a readout might fully replicate their results in the context of arsenite induced SGs (Fig 5).
We agree there are different ways to analyze the data. Kruse et al. studied N-mediated inhibition of the arsenite-induced ISR. The fact that there is a measurable, reproducible but exceedingly subtle effect (see for example also our **Fig. 5**) may have persuaded the authors to use more sensitive methods like counting the SGs and measuring the intensity of G3BP1 foci. Please note, however, that the effects of PKR-induced stress measured in our study are very robust, resulting in large differences between stressed and non-stressed cells such that counting SG-positive cells gives a virtually binary outcome (e.g. see **Figs. 1B**, **1E**, **2A** and all other bar graphs presented). It should also be taking into account that SG formation is only one variable in our assays in addition to i. EGFP fluorescence intensity as measured by IFA and flow cytometry as a measure for translational arrest or inhibition thereof, ii. activation of PKR and iii. phosphorylation of eIF2α (newly added). Our conclusions thus rest on a variety of complimentary observations obtained with a variety of techniques. We do not think reanalyzing our data per the methods of Kruse would change our findings. Moreover, while it may facilitate a comparison with the paper by Kruse et al., it would hamper a comparison with our previous publications in which we developed and validated our experimental approach and successfully applied it to identify new viral ISR inhibitors (Rabouw et al. (2016) PLoS Pathog; Rabouw et al. (2020) Nat Microbiol).

3. In the labeling of the EGFP-viral protein fusion constructs it might be helpful to not only write the fused protein but make clear that its fused to GFP (e.g. instead of SARS-CoV-2 N, EGFP-SARS-CoV-2 N)

Point well taken. We have changed the main text throughout as recommended (e.g. substituting N-EGFP for N where applicable) and the figures as far as their design would allow. Please note that labeling Western blots and bar graphs etc. with the full names in many cases would clutter the Figures and in our opinion would distract from the message rather than inform. In these cases, we have adapted the Legends to convey that transfected cells expressed EGFP-tagged N proteins.

4. It would add to the argument to not only see GFP and SG levels as an ISR readout but also p-PKR levels in Fig 2 and 3.

We already show PKR activation in our expression system upon transfection with the pEGFP vector, and inhibition thereof upon expression of N-EGFP fusion proteins (**Fig. 1**). We also extend the results of **Fig. 2** and **Fig. 3** in panel **Fig. 4C**, where we show that mutations in the N2b domain that abrogate RNA binding also abolish inhibition of PKR activation. In the same panel, we show that suppression of PKR phosphorylation is not affected by mutations in the N1a G3BP binding motif. Finally, we corroborate these observations in EMCV infection experiments (please see **Fig. 7D,** where we again show PKR activation or lack thereof). We consider the evidence provided to be conclusive and do not think that extending the PKR analyses to the experiments in **Figs. 2** and **3** would add additional information.

**Reviewer #3:**

L123-125 - “Transfection of cells with specific expression plasmids like pEGFP-N3 triggers the ISR through dsRNA-mediated PKR activation, resulting in translation arrest and the formation of SGs.” As written, it suggests that transfection of DNA induces the ISR. Why this plasmid induces the ISR should be explained. Is it due to RNA stem loops in the mRNA NCRs present in this plasmid which would be present as part of the mRNAs transcribed for any inserted coding region or to stem loops in the EGFP coding region?

To clarify the mechanism of plasmid-induced activation of the ISR with ensuing formation of SGs, we expanded the text on l. 123-132 with reference to additional publications to read:

*‘Transfection of cells with specific expression plasmids like pEGFP-N3 triggers the ISR through dsRNA-mediated PKR activation, resulting in translation arrest and the formation of SGs (****Fig. 1A****) [27, 41-43]. The dsRNA arises from spurious bidirectional transcription of plasmid sequences [44] and can be readily detected in transfected cells with dsRNA-specific antibodies [27]. This phenomenon allows for a convenient method to identify potential viral ISR antagonists by transiently expressing these proteins genetically fused to enhanced green fluorescent protein (EGFP) [27, 31]. The expression levels of these fusion proteins, as judged by fluorescence microscopy, can then be compared to that of EGFP alone as an indicator for translation arrest and the prevention thereof.’*

We trust that with this additional explanation combined with the visual representation in Fig. 1A and the literature cited most of our readers will grasp the experimental approach.

L364- “transfection of ISR-inducing plasmid DNA” same concern

We now cite literature describing the development and validation of the methodology and have rephrased the text (l. 374-376) to read: ‘*Here we confirmed and extended these observations using a well-established plasmid-based expression system [27, 31] to induce PKR-mediated ISR activation and to assess inhibition thereof by EGFP-tagged ISR antagonists.*’

S Fig 1- The weak signal of the G3BP1 antibody should be acknowledged and used to justify why the rest of the figures have only panels with G3BP2 antibody. A G3BP-1 antibody apparently worked well for IP.

We do not find the staining of SGs by G3BP1 particularly weak (please see below, taken from what is now **Fig. S2**). The antibodies against G3BP2 are of rabbit rather than of murine origin, facilitating double staining with mouse monoclonal antibodies, and therefore, were used throughout for IFA. Please note that the SGs induced by pEGFP-transfection stain positive for G3BP1, G3BP2, eIF3 and TIA1 (see newly added **Fig. S1A** and our reply to Reviewer 2). Staining for both G3BP1 and G3BP2 routinely was deemed non-informative, because these proteins are structurally and functionally closely related and both can associate with the SARS-CoV-2 N protein via the binding motif in domain N1a. In this regard,eIF3 is a better candidate for independent confirmation of observations obtained with G3BP2 as a marker. Indeed, for key experiments staining for G3BP2 and eIF3 were performed in parallel.

S Fig. 2A, Fig 3 etc - EGFP should be used consistently for figure labels not GFP.

Corrected.

S Fig. 2A- “Cell counts” should be added as the label to the Y axis. The vertical dotted line should be explained in the legend.

The text of former S Fig. 2A, now **Fig. S4** has been clarified by adding: ‘*The dashed line marks maximum fluorescence intensity observed in pEGFP-transfected, HeLa wt cells.’*

S Fig. 2- legend- fuorescence is misspelled (ii) “in result” should be changed to “as a result”
Corrected.

S Fig. 3- The EGFP-N fusion protein is what is in the foci in some cells. Legend- …show G3BP2 aggregates that are not positive …

Upon re-reading the Legend to SFig. 3 (**Fig. S5** in the current manuscript), we found it lacking in clarity. This may well have confused the Reviewer for which we apologize. The Legend has been rewritten to read:

‘***Fig. S5*** *(related to Fig. 4)****. Mutagenesis of the serine rich region (SR) of SARS-COV-2 N alters the cellular distribution of N but does not affect its ability to inhibit ISR-induced translation arrest.*** *HeLa R19 cells were transfected to express SARS-CoV-2 N (13S>A)-EGFP. Transfected cells were stained for eIF3 and G3BP2 as markers for SGs. Rather than distributing throughout the cytoplasm, the mutant protein accumulates in large local deposits resembling aggregates. These accumulates contain G3BP2 but in most cases are devoid of eIF3 (cells marked with white crosses). Note that the fluorescence intensity in N (13S>A)-EGFP expressing cells is comparable to that in cells expressing parental N-EGFP (see e.g.* ***Figs. 1****,* ***4****) and consistently higher than in cells expressing EGFP alone or N-N2b mut-EGFP. Size bar: 50µm.*’

L198-201- The meaning of this sentence is not clear as written and needs to be rewritten.
Point well taken. The sentence has been rewritten to read:

*‘An N-EGFP derivative defective in phosphorylation of the SR segment was constructed by replacing 13 out of the 14 Ser residues by Ala. The resulting mutant, N-(13S>A)-EGFP, displayed a cellular distribution distinctively different from that of the parental wildtype protein. Instead of an even distribution throughout the cytoplasm, the mutant protein clustered in what looked like large aggregates. These local deposits were enriched for G3BP2 but mostly devoid of eIF3 (****Fig. 4A****;* ***Fig. S5***). *and differed in size and appearance from typical SGs.’*

Changing all 13 serine residues to alanine could have a major effect of the protein structure but this possibility is not addressed.

We concur but note that the SR segment is part of an intrinsically disordered region with the NTD and CTD folding independently. Admittedly, we do not directly address the possibility of the mutations affecting global protein structure, but do describe the consequences for intracellular distribution, the recruitment of G3BP into large local N accumulates (presumably through N-G3BP interaction) but mostly not so of eIF3. Interesting as these epiphenomena might be, they do not bear on our main observation: the SR and its phosphorylation are NOT essential for N-mediated inhibition of the ISR as judged from the high EGFP fluorescence intensity in N-(13S>A)-EGFP-transfected cells (**Fig. S5**). The results thus provide independent corroboration for the conclusions drawn from the deletion analysis shown in Fig. 2.

L239-240- There are EGFP fusion protein aggregates but they do no seem to be large and also seem to colocalize with G3BP2 (G3BP1 was not tested) but not eIF3 in many transfected cells. However, the eIF3 staining intensity is low and the large white arrows in SFig 3 often cover areas where the foci are located.
The arrows in Fig. S5 (previously Fig. S3) are replaced by white crosses. We do not find the eIF3 staining (now in cyan for reasons of inter-figure consistency) particularly weak. SGs are well discernable as shown here for pEGFP-transfected cells (taken from Fig. S5). For a discussion of the N-EGFP accumulations observed in cells expressing the N-(13S>A)-EGFP mutant, please see below.

There does appear to be colocalization of eIF3 in some of the foci.

We concur. The main text literally reads: ‘…*mostly* devoid of eIF3’

L240-241- No evidence is presented to support the statement that the fusion protein/G3BP2 aggregates differ in size, appearance from SGs.
The evidence is in fact provided in **Fig. 4A**. Stained monolayers were routinely analyzed by the experimentalist (CA) and in blinded fashion by at least one other observer (JGS and/or RdG) and then discussed among the co-authors. The description given in the main text is based on joint assessment. Comparison of the EGFP staining in 13S>A expressing cells to that in other cells (**Fig. 4A**, see for example the panel to the left with cells expressing N-EGFP-4A mut) shows that the N protein is not distributed throughout the cytoplasm but rather seems to concentrate in what looks like large aggregates. These local accumulates also contain G3BP2 as would be expected from N-G3BP2 association. A close examination of G3BP2 staining in 13S>A expressing cells shows a perinuclear aggregated distribution clearly different from the well-defined cytoplasmic granules seen in other cells (Fig. 4A and elsewhere). We considered this an observation worth noting, also because the difference may be evident to attentive readers. However, it is inconsequential to our conclusions, as stated: ‘*We interpret the findings to indicate that the SR mutations may cause the N protein to aggregate but they do not affect inhibition of ISR-induced translational arrest*'.’

L241- …the EGFP expression levels of the ….

L243- …than those of EGFP alone.
For clarity this paragraph (l. 242-250) has been rewritten and corrections were made according to the suggestions of the Reviewer (see also above).

L258- …G3PB1 to a similar extent…
Corrected.

L260- …arrest, the expression levels of these mutants were…
Corrected.

L277- “concentration dependent” It is not clear what data supports this and what is differing in concentration.

Throughout our manuscript, EGFP fluorescence intensity is taken as a measure of intracellular protein concentration. For clarity, however, we altered the text to read: *‘Under these conditions, wt N-EGFP still inhibited SG formation as compared to EGFP alone albeit to a modest extent (****Fig. 5****). This phenomenon may be ascribed to G3BP sequestration, because the observed reduction in the number of SG-producing cells was largely abrogated by mutations in the N1a ФXFG G3BP binding motif* ***(Fig. 5B)****. The inhibition of SG formation seemed dependent on N-EGFP expression levels. The effect was more pronounced in cells with high EGFP fluorescence intensity (****Figs. 5B, 5C; Table S3****).*’

There was no difference detected in the effects of the 4 mutants tested.

That is correct. The four mutants, having one to four substitutions in the N1a ΦXFG G3BP interaction motif, display the same phenotype (actually five, when including the N1a+N1b mutant also shown in **Fig. 5B, C**). In arsenite-treated PKR-KO HeLa cells, the N1a mutant proteins are less able to prevent SG formation than wt N. Essentially, the results obtained with these mutants serve as biological reproductions, reinforcing the notion that the effect observed, modest as it may be, is statistically relevant (**Table S3**).

L340- …delayed to a similar …
Corrected.

L354- “raising the alarm” this is “slang” and too vague

We have altered the text to read: ‘*To successfully establish infection, viruses must subdue the intracellular host defense long enough to complete their replication cycle and vigorously enough to curb intercellular immune signaling.*’

PLOS authors have the option to publish the peer review history of their article (what does this mean?). If published, this will include your full peer review and any attached files.


If you choose “no”, your identity will remain anonymous but your review may still be made public.


**Do you want your identity to be public for this peer review?** For information about this choice, including consent withdrawal, please see our Privacy Policy.

Reviewer #1: No

Reviewer #2: No

Reviewer #3: No

Figure Files:

While revising your submission, please upload your figure files to the Preflight Analysis and Conversion Engine (PACE) digital diagnostic tool, https://pacev2.apexcovantage.com. PACE helps ensure that figures meet PLOS requirements. To use PACE, you must first register as a user. Then, login and navigate to the UPLOAD tab, where you will find detailed instructions on how to use the tool. If you encounter any issues or have any questions when using PACE, please email us at figures@plos.org.

Data Requirements:

Please note that, as a condition of publication, PLOS' data policy requires that you make available all data used to draw the conclusions outlined in your manuscript. Data must be deposited in an appropriate repository, included within the body of the manuscript, or uploaded as supporting information. This includes all numerical values that were used to generate graphs, histograms etc.. For an example see here on PLOS Biology: http://www.plosbiology.org/article/info%3Adoi%2F10.1371%2Fjournal.pbio.1001908#s5.

Reproducibility:

To enhance the reproducibility of your results, we recommend that you deposit your laboratory protocols in protocols.io, where a protocol can be assigned its own identifier (DOI) such that it can be cited independently in the future. Additionally, PLOS ONE offers an option to publish peer-reviewed clinical study protocols. Read more information on sharing protocols at https://plos.org/protocols?utm_medium=editorial-email&utm_source=authorletters&utm_campaign=protocols
